# Sexual Harassment in Academic Medicine in Germany

**DOI:** 10.1001/jamanetworkopen.2025.18237

**Published:** 2025-06-26

**Authors:** Vera Clemens, Marco Kuchenbaur, Claire Richter, Sabine Oertelt-Prigione, Svenja Taubner, Jörg M. Fegert

**Affiliations:** 1 Department of Child and Adolescent Psychiatry, Psychosomatics and Psychotherapy, University Hospital Ulm, Ulm, Germany; 2 German Center for Mental Health, Partner Site Ulm, Ulm, Germany; 3 Medical Didactics and Education Research, Department of Medical Education, Medical Faculty, University of Augsburg, Augsburg, Germany; 4 Sex and Gender Sensitive Medicine, Medical Faculty Ostwestfalen-Lippe, Bielefeld University, Bielefeld, Germany; 5 Department of Primary and Transmural Care, Radboud Universiteit Nijmegen, Nijmegen, the Netherlands; 6 Centre of Psychosocial Medicine, Institute for Psychosocial Prevention, University Hospital Heidelberg, Heidelberg, Germany; 7 German Center for Mental Health, Partner Site Heidelberg, Heidelberg, Germany

## Abstract

This cross-sectional study assesses the prevalence of sexual harassment among physicians and nurses in academic medicine in Germany.

## Introduction

Working in the health sector has an enhanced risk of experiencing sexual harassment.^1^ Although evidence suggests a high prevalence of sexual harassment in health care in Germany,^2^ multicenter studies including nurses are lacking. Consequences of sexual harassment encompass physical, psychological, and work-related problems.^3^ This cross-sectional, large-scale, multicenter study is the first to assess the prevalence of sexual harassment among physicians and nurses in academic medicine in Germany.

## Methods

The study was conducted on a time-delayed basis at the University Hospitals Ulm, Freiburg (March 1 to May 31, 2022), Tübingen (May 20 to August 19, 2022), and Heidelberg (July 13 to October 12, 2022). The anonymous survey was conducted online via Unipark. Recruitment was via email, intranet, flyers, and posters for all employees; only results for physicians and nurses are presented here. Participants provided written informed consent. The survey was approved by the Ethics Commission of Ulm University and followed the STROBE reporting guideline.

Participants were asked if they experienced sexual harassment in the workplace in the last 12 months (eAppendix and eTable in Supplement 1).^2^ With SPSS, version 29.0, χ^2^ tests and multivariate logistic regressions were performed, assessing gender identity, department (predominantly surgical, minor or no surgical component, research, or administration), gender distribution on the team (1 = only women, 10 = only men), hierarchy (1 = flat hierarchy, 10 = strong hierarchy), leadership position (nurses), medical education status (physicians), and gender of team leader. A 2-sided *P* < .05 was considered significant.

## Results

A total of 1499 of 6333 physicians (23.7%) and 2530 of 11 422 nurses (22.2%) participated in the study; 99 physicians and 155 nurses did not provide information on sexual harassment, and 1 nurse did not provide information on gender. Seven physicians and 9 nurses reported their gender as transgender or nonbinary and could not be included in analyses due to the low number and to ensure anonymity. The final sample included 1403 physicians (56.2% women and 43.8% men) and 2365 nurses (81.0% women and 19.0% men).

Together, 74.2% of female physicians, 51.2% of male physicians, 77.1% of female nurses, and 68.2% of male nurses experienced sexual harassment in the workplace, approximately one-third within the last 12 months (Table 1). More women than men perceived the misconduct as harassing and threatening (physicians, 82.8% vs 61.5%; nurses, 84.9% vs 63.8%; *P* < .001).

**Table 1.** Prevalence of Sexual Harassment^a^

Lifetime prevalence	Physicians, No./total No. (%)		*P* value	Nurses, No./total No. (%)		*P* value
	Female	Male		Female	Male	
Sexual harassment						
Any form	585/788 (74.2)	315/615 (51.2)	<.001	1477/1916 (77.1)	306/449 (68.2)	<.001
Sexualized remarks, sexual innuendo	422/788 (53.6)	165/615 (26.9)	<.001	1014/1916 (52.9)	157/449 (35.0)	<.001
Stories with sexual content	281/788 (35.7)	158/615 (25.7)	<.001	842/1916 (43.9)	190/449 (42.3)	.53
Sexual offers, unwanted invitations	149/788 (18.9)	75/615 (12.2)	<.001	504/1916 (26.3)	79/449 (17.6)	<.001
Devaluation, obscenity via telephone, letters, emails, text messages	96/788 (12.2)	113/615 (18.4)	.001	233/1916 (12.2)	78/449 (17.4)	.003
Whistling, staring, undressed with looks	253/788 (32.1)	57/615 (9.3)	<.001	876/1916 (45.7)	71/449 (15.8)	<.001
Obscene gestures and signs	127/788 (16.1)	84/615 (13.7)	.21	573/1916 (29.9)	123/449 (27.4)	.29
Advantages for sexual favors	17/788 (2.2)	9/615 (1.5)	.34	58/1916 (3.0)	8/449 (1.8)	.15
Unwanted physical contact	356/788 (45.2)	141/615 (22.9)	<.001	1052/1916 (54.9)	174/449 (38.8)	<.001
Groping, attempted kissing	86/788 (10.9)	29/615 (4.7)	<.001	390/1916 (20.4)	63/449 (14.0)	.002
Sexual assault	11/788 (1.4)	5/615 (0.8)	.31	81/1916 (4.2)	5/449 (1.1)	.002
Other situations	82/788 (10.4)	33/615 (5.4)	<.001	251/1916 (13.1)	44/449 (9.8)	.06
Perceived the misconduct as harassing^b^	461/557 (82.8)	182/296 (61.5)	<.001	1151/1355 (84.9)	169/265 (63.8)	<.001
Perceived the misconduct as threatening	202/557 (36.3)	51/296 (17.2)	<.001	631/1355 (46.6)	47/265 (17.7)	<.001
12-mo Prevalence of sexual harassment	279/788 (35.4)	166/615 (27.0)	.001	711/1916 (37.1)	160/449 (35.6)	.59
Perpetrators’ gender^b^						
Only or primarily women	1/542 (0.2)	74/282 (26.2)	<.001	13/1305 (1.0)	66/242 (27.3)	<.001
Only or primarily men	507/542 (93.5)	99/282 (35.1)	<.001	1106/1305 (84.8)	51/242 (21.1)	<.001
All genders	34/542 (6.3)	109/282 (38.7)	<.001	186/1305 (14.3)	125/242 (51.7)	<.001
Perpetrators’ occupational group^b^^,^^c^						
Patients	303/544 (55.7)	116/289 (40.1)	<.001	967/1315 (73.5)	171/255 (67.1)	.03
Colleagues	359/544 (66.0)	247/289 (85.5)	<.001	771/1315 (58.6)	177/255 (69.4)	.001
Patients’ relatives	83/544 (15.3)	37/289 (12.8)	.34	285/1315 (21.7)	51/255 (20.0)	.55
Supervisors	238/544 (43.8)	59/289 (20.4)	<.001	181/1315 (13.8)	19/255 (7.5)	.006
Others	37/544 (6.8)	21/289 (7.3)	.79	124/1315 (9.4)	16/255 (6.3)	.11

^a^ All data refer to the lifetime prevalence, unless otherwise stated.

^b^ Only persons who have experienced at least 1 form of sexual harassment were included.

^c^ Multiple answers were accepted.

In total, 93.5% of female physicians and 84.8% of female nurses experienced sexual harassment committed exclusively or primarily by men (*P* < .001) (Table 1). Male physicians experienced sexual harassment committed most frequently by all genders. Female physicians and nurses experienced sexual harassment committed more frequently by patients and male physicians, and nurses experienced sexual harassment committed more frequently by colleagues.

Risk factors for sexual harassment were female gender; age younger than 40 years; working in a clinical department, in particular with a predominantly surgical component; and a stronger hierarchy (Table 2). Among nurses, having a higher proportion of men on the team and a male team leader were associated with sexual harassment.

**Table 2.** Risk Factors Associated With Sexual Harassment^a^

Risk factor	Physicians		Nurses	
	AOR (95% CI)	*P* value	AOR (95% CI)	*P* value
**Lifetime prevalence**				
Female gender	3.13 (2.04-4.08)	<.001	1.64 (1.24-2.17)	<.001
Age <40 y	1.14 (0.82-1.60)	.44	1.96 (1.57-2.46)	<.001
Department with a predominantly surgical component	2.49 (1.55-4.02)	<.001	1.38 (1.06-1.78)	.02
Department with minor or no surgical component (eg, internal medicine)	1.76 (1.14-2.72)	.01	1.55 (1.20-2.00)	<.001
Gender distribution of team	1.05 (0.96-1.16)	.29	1.15 (1.06-1.26)	.002
Hierarchy	1.19 (1.12-1.26)	<.001	1.14 (1.08-1.21)	<.001
Leadership position (nursing staff)	NA	NA	1.35 (1.00-1.83)	.05
Resident physicians	1.06 (0.76-1.48)	.72	NA	NA
Male team leader	1.26 (0.94-1.69)	.12	1.22 (0.93-1.59)	.15
No.	1166	NA	1829	NA
Nagelkerke *R*^2^	0.168	NA	0.077	NA
**12-mo Prevalence**				
Female gender	1.55 (1.18-2.03)	.002	0.97 (0.74-1.27)	.84
Age <40 y	2.23 (1.55 3.21)	<.001	3.43 (2.77-4.25)	<.001
Department with a predominantly surgical component	2.07 (1.23-3.48)	.006	1.18 (0.93-1.49)	.18
Department with minor or no surgical component (eg, internal medicine)	1.26 (0.77-2.07)	.36	0.96 (0.76-1.21)	.70
Gender distribution of team	1.06 (0.96-1.16)	.24	1.15 (1.06-1.24)	<.001
Hierarchy	1.13 (1.06-1.20)	<.001	1.05 (1.00-1.10)	.05
Leadership position (nursing staff)	NA	NA	0.99 (0.75-1.31)	.95
Resident physicians	1.14 (0.83-1.58)	.42	NA	NA
Male team leader	1.11 (0.82-1.52)	.51	1.29 (1.02-1.63)	.04
No.	1166	NA	1829	NA
Nagelkerke *R*^2^	0.130	NA	0.118	NA

Abbreviations: AOR, adjusted odds ratio; NA, not applicable.

^a^ Multivariate logistic regression analyses. Male gender is the reference category for female gender; department with minor or no surgical component (eg, internal medicine), research, or administration is the reference category for department with a predominantly surgical component; department with a predominantly surgical component, research, or administration is the reference category for department with no or low surgical component (eg, internal medicine); gender distribution of team (1 = only women, 10 = only men; hierarchy: 1 = flat hierarchy, 10 = strong hierarchy); no leadership position is the reference category for leadership position; fellow, attending, and chief physician is the reference category for resident physicians; and female manager or shared manager (man and woman) is the reference category for male gender of team leader.

## Discussion

Results are comparable to prevalence rates of sexual harassment found in national and international surveys in academic settings,^2,4^ although others found lower prevalence rates.^5^ This may because we used specific situations in our survey instead of a general question on sexual harassment, which could lead to an underestimation through differing perceptions of sexual harassment.^6^

Female gender and male-dominated structures have been associated with sexual harassment.^1,2^ Our results also indicate a high prevalence of sexual harassment among men. Younger age and working in clinical departments, particularly with surgical components, are well-known risk factors for sexual harassment,^1,2^ indicating a need for targeted protective measures for this group. Stronger hierarchy, along with a power gradient that can favor abuse of power, is associated with increased risk for sexual harassment.^1^

Limitations comprise the lack of representativeness of the sample and selection of participants due to this sensitive topic. Sexual harassment is a cultural problem, affecting women and men, and physicians and nurses, and must be addressed by systemwide changes, valuing and supporting diversity, inclusion, and respect.^1^

## Notes

Supplement 1. **eTable.** Response Rates **eAppendix.** Questionnaire

Supplement 2.
Data Sharing Statement
