# Supplementary material for: Sexual Harassment in Academic Medicine in Germany
Source: JAMA Netw Open. 2025 Jun 26;8(6):e2518237. doi: 10.1001/jamanetworkopen.2025.18237 (PMC12203272; doi:10.1001/jamanetworkopen.2025.18237)
Supplement: Supplement 1. — eTable. Response Rates eAppendix. Questionnaire [file jamanetwopen-e2518237-s001.pdf]

Supplementary Online Content

Clemens V, Kuchenbaur M, Richter C, Oertelt-Prigione S, Taubner S, Fegert JM. Sexual harassment in academic medicine in Germany. *JAMA Netw Open.* 2025;8(6):e2518237. doi:10.1001/jamanetworkopen.2025.18237

eTable. Response Rates

eAppendix. Questionnaire

This supplementary material has been provided by the authors to give readers additional information about their work.

© 2025 Clemens V et al. *JAMA Network Open.*

eTable. Response Rates

	University Hospital Ulm			University Hospital Freiburg			University Hospital Tübingen			University Hospital Heidelberg			Total		
	Number of Participants (N)	Number of employees (N)	Response rate (%)	Number of Participants (N)	Number of employees (N)	Response rate (%)	Number of Participants (N)	Number of employees (N)	Response rate (%)	Number of Participants (N)	Number of employees (N)	Response rate (%)	Number of Participants (N)	Number of employees (N)	Response rate (%)
Physicians	346	971	35.6 %	291	1828	15.9 %	338	1441	23.5 %	524	2093	25.0 %	1499	6333	23.7 %
Nurses	550	1712	32.1 %	768	3472	22.1 %	639	2740	23.3 %	573	3498	16.4 %	2530	11422	22.1 %

© 2025 Clemens V et al. *JAMA Network Open.*

eAppendix. Questionnaire

Have you experienced the following harassment in the workplace? (Please tick all that apply)

A) Someone speaks degradingly of women, men, trans*people or homosexuals or makes obscene jokes.[1]

B) Someone has sent you degradingly or obscene jokes and sayings, pornographic or nude pictures by telephone, letter, e-mail or mobile phone.

C) Someone has made sexual remarks about you, your appearance, your clothing or sexual innuendos or derogatory remarks about you.

D) Someone has whistled at you in an unwanted way, stared at you indecently or ‘undressed you with looks’.

E) Someone has made obscene gestures or signs towards you.

F) Someone has told you unwanted stories with sexual content or had conversations with you with sexual content.

G) Someone has made you intrusive sexual offers or unwanted invitations with sexual intentions.

H) Someone has made unwanted physical contact through seemingly accidental touching or physically unnecessary proximity.

I) Someone has groped you or tried to kiss you against your will.

J) Someone has threatened you with advantages in case of sexual favour or disadvantages in case of refusal.

K) Someone has sexually assaulted you.

L) Other situations…………

▢ ever in the course of your entire professional activity

▢ during the last 12 months

▢ no

[1] Due to editorial consideration, this item was not included into the prevalence of sexual harassment.

© 2025 Clemens V et al. *JAMA Network Open.*
