# Supplementary material for: Sexual Harassment in Academic Medicine in Germany
Source: JAMA Netw Open. 2025 Jun 26;8(6):e2518237. doi: 10.1001/jamanetworkopen.2025.18237 (PMC12203272; doi:10.1001/jamanetworkopen.2025.18237)
Supplement: Supplement 2. — Data Sharing Statement [file jamanetwopen-e2518237-s002.pdf]

# Data Sharing Statement

Clemens. Sexual Harassment in Academic Medicine in Germany. *JAMA Netw Open*. Published June 26, 2025. doi:10.1001/jamanetworkopen.2025.18237

**Data**
**Data available:** Yes
**Data types:** Other (please specify)
**Additional Information:** Data will be provided on reasonable request.
**How to access data:** vera.clemens@uni-ulm.de
**When available:** With publication

**Supporting Documents**
**Document types:** None

**Additional Information**
**Who can access the data:** Data will be provided on reasonable request for researchers whose proposed use of the data has been approved.
**Types of analyses:** Data dictionary
**Mechanisms of data availability:** With a signed data access agreement
